# Microbial Production of Violacein and Process Optimization for Dyeing Polyamide Fabrics With Acquired Antimicrobial Properties

**DOI:** 10.3389/fmicb.2018.01495

**Published:** 2018-07-10

**Authors:** Maria Kanelli, Mina Mandic, Margarita Kalakona, Sozon Vasilakos, Dimitris Kekos, Jasmina Nikodinovic-Runic, Evangelos Topakas

**Affiliations:** ^1^IndBioCat Group, Biotechnology Laboratory, School of Chemical Engineering, National Technical University of Athens, Athens, Greece; ^2^Institute of Molecular Genetics and Genetic Engineering, University of Belgrade, Belgrade, Serbia; ^3^Materials Industrial Research and Technology Center S.A., Athens, Greece; ^4^Biochemical and Chemical Process Engineering, Division of Sustainable Process Engineering, Department of Civil, Environmental and Natural Resources Engineering, Luleå University of Technology, Luleå, Sweden

**Keywords:** violacein, bacterial pigment, *Janthinobacterium lividum*, polyamide, antimicrobial activity

## Abstract

In the present study, crude bacterial extract containing violacein is investigated for the preparation of antimicrobial polyamide fabrics. The optimal culture conditions of *Janthinobacterium lividum* (JL) for maximum biomass and violacein production were found to be 25°C, pH 7.0, while the addition of ampicillin of 0.2 mg mL^-1^ in the small scale increased violacein production 1.3-fold. In scale-up trials, the addition of 1% (v/v) glycerol in a fed-batch bioreactor, resulted in fivefold extracted crude violacein increase with final concentration of 1.828 g L^-1^. Polyamide 6.6 fabrics were dyed following three different processes; through simultaneous fermentation and dyeing (SFD), by incubating the fabric in the sonicated bacterial culture after fermentation and by using cell-free extract containing violacein. Maximum color change (Δ*E*) and color strength (*K*/*S*) obtained for SFD fabrics were 74.81 and 22.01, respectively, while no alteration of fastness and staining of dye at acid and alkaline perspiration or at water was indicated. The dyed fabrics presented significant antifungal activity against *Candida albicans, C. parapsilosis*, and *C. krusei*, as well as antibacterial properties against *Escherichia coli, Staphylococcus aureus*, and the *S. aureus* MRSA. We have shown that *J. lividum* cultures can be successfully used for violacein production and for simultaneous dying of fabrics resulting in dyed fabrics with antimicrobial properties without utilization of organic solvents.

## Introduction

Over the years, the production of natural pigments from different biological sources, such as bacteria, fungi, and algae has been established. While synthetic dyes of lower cost rapidly expanded in different applications including textile industry, it remains an undisputable fact that biological dyes remain a “green” solution due to their biodegradability. Moreover, the biotechnological dye production through fermentation processes comprises an eco-friendly approach, without the drawbacks that the chemical synthesis of dyes present.

Additionally, the modification of fabrics in order to acquire antimicrobial properties and other functionalities represents a new trend because of the “superbugs” problem (Cerkez et al., 2013; Yu et al., 2016; Alpert, 2017). In this unprecedented situation, several microorganisms maleficent or noxious for human health, such as *Clostridium difficile* and *Enterobacteriaceae*, have evolved and developed multidrug resistance (Aruguete et al., 2013; Alpert, 2017). In literature, the surface grafting of cotton fabrics with irradiation and 1-butyl-3-vinyl imidazole chloride bonding has been reported for antibacterial use against superbugs, such as methicillin-resistant *Staphylococcus aureus*, vancomycin-resistant *Enterococcus faecium, Acinetobacter calcoaceticus*, and other common bacteria (Yu et al., 2016). Turning to a biotechnological direction, fabric dyeing using biological dyes with antimicrobial properties, such as prodigiosins (Stankovic et al., 2014) or violacein, is an attractive prospective to address the arising health problem.

Violacein [3-(1,2-dihydro-5-(5-hydroxy-1*H*-indol-3-yl)-2- *oxo*-3*H*-pyrrol-3-ilydene)-1,3-dihydro-2*H*-indol-2-one], is an indole derivative compound with a deep purple hue. It is produced from various bacteria such as *Pseudoalteromonas luteoviolacea, Alteromonas luteoviolacea*, and *Chromobacterium violaceum*, including *Janthinobacterium lividum* (JL) (Laatsch et al., 1984; Yada et al., 2008; Rodrigues et al., 2012; Rahul et al., 2015) and specifically the 1522 strain (ATCC 12473; Kimmel and Maier, 1969) that was used in the present study. Recently, the draft genome sequence of two *J. lividum* strains was published, revealing the biosynthesis and regulation of violacein production (Wu et al., 2017). Depending on the strain, the culture medium, and conditions of growth a different path is followed for violacein production. The production of this purple pigment in microorganisms possibly represents a response to environmental stresses and contributes to the microorganism’s defense against external danger (Pantanella et al., 2007).

Violacein’s production from various bacteria and its notable properties have been studied thoroughly before. Violacein has been proved to have antimicrobial, antileishmanial, antiviral, as well as antitumor properties (Leon et al., 2001; Nakamura et al., 2003; Kodach et al., 2006; Masuelli et al., 2016). The antimicrobial impact of violacein from *Duganella violaceinigra* strain NI28 was recently justified against *S. aureus* (Choi et al., 2015a). Additionally, violacein acts against phytopathogenic fungi such as *Rosellinia necatrix* (Durán et al., 2007). It also exhibited antiprotozoal activity toward nanoflagellates (Matz et al., 2004). The pro-apoptotic activity of violacein from *C. violaceum* CCT 3496 was demonstrated *in vivo* and *in vitro* in Ehrlich ascites tumor and in MOLT-4 leukemia (Melo et al., 2000; Bromberg et al., 2010). Recently the potential use of violacein in sunscreens has been suggested and investigated (Suryawanshi et al., 2015). Violacein has also been used as a colorant for a variety of natural and synthetic fabrics (Shirata et al., 2000) instead of other chemical colorants for textile dyeing (Kanelli et al., 2017).

In the present study, the potential use of this purple pigment for dyeing synthetic textiles is examined with the ulterior purpose to produce materials with acquired antimicrobial and antioxidant activity for application in a variety of fields, such as medicinal or cleansing clothing or bags and other packaging applications. Initially, a culture optimization study is conducted in order to maximize biomass growth and violacein production. Scale-up batch and fed-batch cultures in bioreactor were performed to increase the amount of violacein production for dyeing textiles. Polyamide 6.6 fabrics were dyed via simultaneous fermentation and dyeing (SFD) or direct dyeing (DD) of fabrics with cell-free extract containing violacein or by incubation in the supernatant after fermentation and sonication of the culture cells (DAFS). Color fastness and staining of dye at acid and alkaline perspiration were evaluated in the final dyed textiles. Antimicrobial and antioxidant potential of the dyed fabrics was also investigated in order to evaluate the added value of biotechnological process proposed in this investigation.

## Materials and Methods

### Materials and Chemicals

Nutrient broth (NB) consisting of 3 g L^-1^ meat extract, 5 g L^-1^ peptone and salts was used as a medium for bacterial cultures. Nutrient agar petri-dishes were prepared with composition 3 g L^-1^ meat extract, 5 g L^-1^ peptone, and 15 g L^-1^ agar. Sabouraud medium was used for maintenance of *Candida* spp. and it contained 40 g L^-1^ glucose, 10 g L^-1^ peptone, 20 g L^-1^ agar, pH 5.6.

1,1-Diphenyl-2-picryl-hydrazyl (DPPH) was purchased from Sigma (St. Louis, MO, United States). Methanol used was of HPLC grade. Polyamide knitted fabric with density 23 columns cm^-1^ and 15 rows cm^-1^, weight 12 g m^-2^, 154.7 Denier and average thickness 0.309 mm was supplied by Colora S.A. (Thessaloniki, Greece). The PA fabric was washed with detergent Felosan NFG before use for the removal of impurities.

### Microorganisms

*Janthinobacterium lividum* 1522 strain ATCC 12473 was provided by WAAG Society, TextileLab, Amsterdam (Netherlands) and was maintained frozen in Luria Bertani (LB) broth containing glycerol 20% (v/v) at -80°C. Strains used to determine antimicrobial properties, *Pseudomonas aeruginosa* PAO1 ATCC 47085, *Escherichia coli* ATCC 25922, *S. aureus* ATCC 25923, *S. aureus* MRSA ATCC 43300, *Micrococcus luteus* ATCC 379, *Bacillus subtilis* ATCC 6633, *Candida albicans* ATCC 10231, *C. krusei* ATCC 6258, and *C. parapsilosis* ATCC 22019, were obtained from the American Type Culture Collection (ATCC).

### Optimization of Violacein Production and Extraction

Nutrient agar petri-dishes were prepared prior to their inoculation with *J. lividum* stock at -80°C and were incubated at 25°C overnight. Subsequently, 20 mL of NB in 100 mL Erlenmeyer flasks were inoculated with *J. lividum* and incubated at 25°C, under agitation (180 rpm). A final volume of 1% inoculum was transferred into 250 mL Erlenmeyer flasks containing 100 mL of fresh NB when the pre-culture’s cell concentration reached the optical density OD_600_ value of 3. Several conditions that affect violacein production were examined in order to optimize its production. A broad range of initial pH (5.0–9.0) was studied using the following buffer systems to achieve the desired pH: 15.6 g L^-1^ NaH_2_PO_4_⋅2H_2_O for pH 5.0, 13.25 g L^-1^ NaH_2_PO_4_⋅2H_2_O and 2.67 g L^-1^ Na_2_HPO_4_⋅2H_2_O for pH 6.0, 5.72 g L^-1^ NaH_2_PO_4_⋅2H_2_O and 11.3 g L^-1^ Na_2_HPO_4_⋅2H_2_O for pH 7.0, 0.85 g L^-1^ NaH_2_PO_4_⋅2H_2_O and 16.8 g L^-1^ Na_2_HPO_4_⋅2H_2_O for pH 8.0 and 17.8 g L^-1^ Na_2_HPO_4_⋅2H_2_O and 10% w/v Na_2_CO_3_ for pH 9.0. The effect of temperature was also studied for violacein production in the range of 20–35°C. Biomass and violacein production were also examined in NB medium in the presence of glucose (NB-G), glycerol (NB-GY), or xylose (NB-X) at the concentration of 1% (w/v). Subsequently, the addition of 0.1 mg mL^-1^ of antibiotic was examined. The antibiotics tested were ampicillin, kanamycin, streptomycin, and tetracycline. Finally, the effect of ampicillin concentration in the culture supernatant was tested in the range between 0.05 and 0.4 mg mL^-1^. All culture conditions were carried out at 180 rpm and in duplicate.

Biomass production was measured daily on a dry weight (d.w.) basis. Crude violacein was cell extracted from sample volume with methanol (2.0:1.5, v/v), after incubation 150 rpm, for 15 min at room temperature and quantified by measuring the absorbance at 580 nm on a BOECO S-20 Spectrophotometer. The gravimetric yield of cell-free extract containing violacein from biomass at optimal growth conditions of temperature and pH was estimated. Additionally, the amount of violacein from the supernatant was also measured at 580 nm and it was extracted with ethyl acetate.

### Production of Violacein in Bioreactor

Cultures of 50 in 250 mL Erlenmeyer flasks were inoculated with *J. lividum* and incubated at 25°C, under agitation (180 rpm), overnight. An inoculum was transferred into 1.5 L of fresh NB pH 7 so that biomass concentration reached the OD_600_ value of 0.2. The scale-up bacterial culture was conducted in 2 L capacity Bioflo^®^ and Celligen^®^ 310 Fermentor (New Brunswick Scientific, Edison, NJ, United States). In addition, a fed-batch culture was studied, where at the logarithmic growth phase where OD_600nm_ was equal to 1.7, glycerol of final concentration 1% (v/v) was added in order to boost the biomass production and eventually violacein formation. The temperature was controlled at 25°C and the pH of the culture was controlled at 7.0 using a solution of 2M HCl. Ventilation was fixed at 0.75 vvm, with the dissolved oxygen level controlled above 20 ± 10% with Sen, Inpro 6830/12/220/NBS O_2_ sensor (Mettler Toledo). Samples were withdrawn during fermentation to measure biomass production via optical density at 600 nm and on d.w. basis, violacein production and glycerol consumption.

Cell biomass was freeze dried and crude violacein was extracted with methanol, after incubation for 30 min at room temperature and 150 rpm. Methanol was evaporated and the cell-free extract containing violacein was stored in dark at 4°C. For the estimation of violacein content in the extract, violacein was dissolved in ethanol and the extinction coefficient of 56.01 mL mg^-1^ cm^-1^ at 575 nm was applied, which corresponds to pure violacein in ethanol solution (Mendes et al., 2001).

### Dyeing of Polyamide Fabrics With Violacein

Polyamide 6.6 samples were dyed following three different processes; SFD where the fabric is incubated simultaneously with the bacterium, DAFS where fabric was dyed using the culture supernatant that was enriched by violacein released after disrupting the cells with sonication, and DD (with cell-free extract) where cell-free extract containing violacein from bacterial biomass was utilized directly.

In SFD, sterilized fabrics (5 cm × 5 cm) were incubated with *J. lividum* under optimal growth conditions of pH and temperature in 250 mL Erlenmeyer flasks for 6 days. After incubation, fabric samples were autoclaved and washed with 0.4% Triton X-100 for 1 h at 80°C and finally with distilled water.

In DAFS process, after fermentation of *J. lividum* for 6 days, the culture underwent sonication (40% amplitude 20 min pulse 2 min On, 2 min Off) for maximizing violacein release. The cell debris was removed and fabric sample 5 cm × 5 cm was incubated for 48 h after the addition of 0.02% (w/v) sodium azide. Dyed fabrics were autoclaved and washed with 0.4% Triton X-100 for 1 h at 80°C and finally with distilled water. All studies were performed in duplicate.

In DD polyamide fabrics were dyed with the cell-free extract containing violacein from cultures that were incubated for 6 days, fabric sample 5 cm × 5 cm was incubated in 100 mL Erlenmeyer flasks containing 30 mL of cell-free extract containing violacein (1.02 mg mL^-1^) in methanol:water 1.7:10.0 (v/v) for 48 h. The dyed samples were washed with distilled water.

### Analysis of Violacein Dyed Textiles

Color coordinates were evaluated according to CIE *L*^∗^*a*^∗^*b*^∗^ System, established by the “Commission Internationale de l’ Eclairage–CIE” according to ASTM D 2244-68, with the use of a color tris-timulus colorimeter (Data Color International, Spectraflash SF450) (Lawrenceville, NJ, United States). For each sample, four repeated measurements were taken to determine color coordinates *L, a, b*. Color change (Δ*E*) and color strength (*K*/*S*) were calculated as described before (Kanelli et al., 2015).

A crockmeter equipment was used to determined abrasion resistance in dry and wet state of polyamide dyed fabric according to EN ISO 105-X12. In the movable place of the equipment a white dry or wet cotton fabric was fitted and was rubbed on the sample of the dyed fabric by performing certain cycles and under certain pressure. After test completion, the white cloth was removed and evaluated for staining on a gray scale from 1 to 5 (where 5 is regarded as no staining). Similarly, wet white cotton fabric was fitted and tested in order to determine color fastness on it.

Color fastness and staining of dye at acid and alkaline perspiration were measured according to EN ISO 105 E04 and color fastness and staining of dye at water according EN ISO 105 E01. Fabric specimens were fully wet at acid, alkaline and water solution and after 30 min were placed between specific pressure plates, and remained there for 4 h at 37°C (simulating of human temperature). Dyed polyamide fabric was sewed with standard control fabric comprising control zones with the following compositions: wool, acrylic, polyester, nylon, cotton, acetate, and silk. The assessment of the specimens took place according to the gray scales of staining and Δ*E*.

Furthermore, the color fastness to artificial light was assessed with XENON arc fading lamp test. In order to determine the color fastness after exposure to artificial light, specimens were placed in a XENON arc fading lamp chamber and tested according to EN ISO 105-B02:2013, with temperature of 45°C, humidity 40% and irradiance 42 W m^-2^ in a wavelength of 300–400 nm.

### Antimicrobial Activity Tests

Antimicrobial activity of polyamide fabric was quantitatively tested in liquid NB culture on a panel of bacterial strains*: E. coli, P. aeruginosa, S. aureus, S. aureus* MRSA, *B. subtilis*, and *M. luteus* using previously published procedure based on American Association of Textile Chemists and Colorists AATCC 100-1993 method (Shahmoradi Ghaheh et al., 2014). Antifungal activity was tested on *C. albicans, C. krusei*, and *C. parapsilosis* using the same approach. Briefly, overnight culture of each test organism was diluted in 2 mL NB medium to a final optical density, OD_600_, of 0.1. Fabric samples of 100 mg mL^-1^ were sterilized by briefly wiping with 70% ethanol (v/v). Alternatively, methanolic extracts containing violacein were dissolved in DMSO (50 mg mL^-1^, stock solutions) and were added to cultures in final concentrations of 2, 1, 0.5, and 0.1 mg mL^-1^. Tubes were incubated at 37°C for 24 h with shaking at 180 rpm. Serial dilutions were prepared and plated onto NB agar medium. Colony forming units (c.f.u.) were enumerated after 24 h. The percentage of reduction in the number of bacteria was calculated by the following equation:

$$\begin{array}{c} R(\%)=\frac{A-B}{A}*100, \end{array}$$

where *R* is the percentage reduction of bacteria, *A* represents the number of bacteria colonies in the control (the untreated fabric), and *B* represents the number of bacteria colonies in the treated fabrics. Appropriate controls, such as medium with or without polyamide samples were included in each experiment. Tests were performed in duplicate in two independent experiments.

### Antibiofilm Assays

Ability of *J. lividum* crude methanolic extracts containing violacein to inhibit the formation and to disperse preformed biofilms of *S. aureus* ATCC 25923 and *C. albicans* ATCC 10231 were tested in 96-well microtiter format using a crystal violet (CV) method for staining the adherent cells as described previously (Merritt et al., 2005; Pierce et al., 2010). Experiments were performed in hexaplicates using the methanolic extract containing violacein dissolved in DMSO (50 mg mL^-1^, stock solution) or DMSO as a control. Final concentrations of extracts were 1, 0.5, and 0.25 mg mL^-1^ for monitoring the inhibition of biofilm formations 2, 1, and 0.5 mg mL^-1^ for dispersion of preformed biofilms. Briefly, overnight culture of *S. aureus* was diluted in LB to 3 × 10^7^ cfu mL^-1^, while *C. albicans* overnight culture was diluted in RPMI medium supplemented with 2% (w/v) glucose to 2 × 10^6^ cfu mL^-1^. Biofilms were formed in the presence of the extract containing violacein for 24 h (*S. aureus*) and 48 h (*C. albicans*) at 37°C under static conditions. In the biofilm dispersion assay, the extract was added to already formed biofilms and incubated for further 24 h. In both experiments, biofilms were washed with phosphate saline buffer (PBS), and adherent cells were stained with 0.1% (v/v) CV. Optical density of formed biofilms and biofilms that remained after treatment was measured at a wavelength of 590 nm.

### Antioxidant Potential of Violacein Dyed Fabrics

Fabric samples (2.5 cm × 2.5 cm) were incubated in 5 mL of DPPH free radical solution in ethanol (5.07 × 10^-5^ M) for 1 h at room temperature in the dark. The antioxidant surface reacts with the stable free radical of DPPH producing a colorless DPPH, as reported elsewhere (Konzen et al., 2006). The absorbance at 517 nm was measured using a BOECO S-20 Spectrophotometer against a blank without fabric. All analyses were carried out in duplicate. The scavenging ability was calculated by the following equation:

$\mathrm{Scavenging}\;\mathrm{ability}\;\%=\left(1-\left(\frac{{\mathrm{Abs}}_{\mathrm{sample}}}{{\mathrm{Abs}}_{\mathrm{blank}}}\right)\right)•100$; where blank is the DPPH radical solution in ethanol.

## Results

### Optimization of Culture Conditions for Violacein Production

**FIGURE 1:**
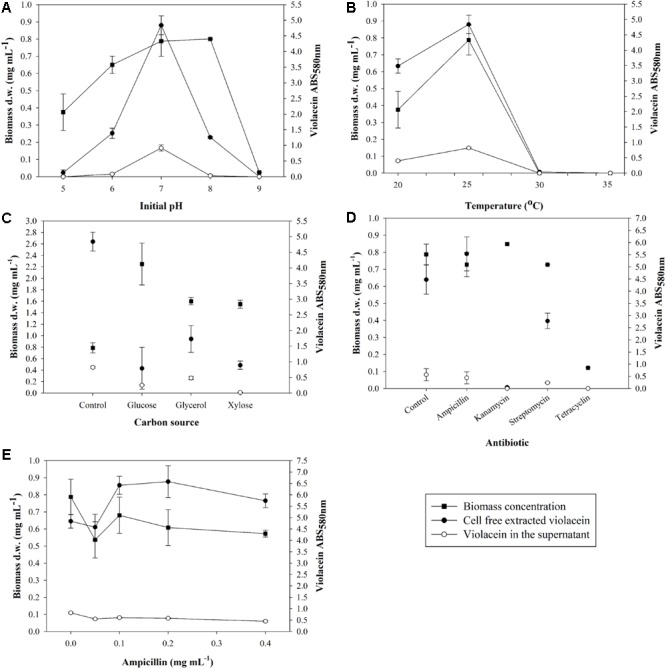
Effect of **(A)** initial pH, **(B)** temperature, **(C)** carbon source, **(D)** antibiotics, and **(E)** ampicillin on biomass concentration of *J. lividum* and crude violacein production cell-free extracted and leaked in the supernatant.

Initially, an optimization study was conducted aiming at the optimal conditions that boost biomass production along with violacein production. The effect of an initial pH range from 5.0 to 9.0 was examined, as shown in **Figure 1A**. Biomass was maximized for initial pH 7.0 and 8.0, however, violacein production was up to 3.8-fold higher for initial pH 7.0 comparing to pH 6.0 and 8.0. On the other hand, lower production of biomass was observed for pH 5.0 with no violacein production, where pH 9.0 proved a hostile environment for *J. lividum* prosperity.

Subsequently, the effect of fermentation temperature in the range between 20 and 35°C was examined (**Figure 1B**). Psychrotrophic microorganisms attain a relatively lower optimum temperature of growth due to the thermolability of one or more essential cellular components, particularly enzymes (Gounot, 1986). *J. lividum* seemed to grow well in the vicinity of 20–25°C, where 30 and 35°C were considered an inhibitory temperature. Maximum biomass was produced at 25°C with average concentration 0.79 mg mL^-1^ accompanied by maximum pigment production. At 25°C and initial pH 7.0, average violacein production after 6 days of incubation in 100 mL culture was measured to be 0.305 mg mL^-1^. Furthermore, the leaked violacein in the supernatant was measured to be 0.215 mg mL^-1^, indicating a loss of 41% of the total amount of violacein that is produced in the cells. At higher temperatures of 30 and 35°C no biomass production was noted and subsequently there was no violacein production, while at 20°C biomass reached a concentration of 0.38 mg mL^-1^ with second best violacein production, justifying the psychrotrophic character of *J. lividum*.

In a following step, the carbon source context in the *J. lividum* cultures was assessed. Glucose, glycerol and xylose were chosen as the most common and less expensive carbon sources for microorganism nutrition. All three media NB-G, NB-GY, NB-X had a positive impact on biomass production, however, violacein output does not show a similar trend (**Figure 1C**). NB-G accomplished the highest biomass concentration of 2.25 mg mL^-1^, nonetheless, it inhibited violacein production as opposed to basic NB as medium. NB-GY and NB-X also led to higher biomass production (1.60 and 1.55 mg mL^-1^, respectively) but lower than NB-G. Violacein production proved to maximize in NB, while NB-GY presented the second best results in deriving the target metabolite.

Finally, the effect on violacein production was examined by adding different antibiotics. **Figure 1D** proves that only the addition of ampicillin to the culture of *J. lividum* improves violacein production without acute influence on biomass growth. It is assumed that ampicillin addition represents stress-inducing factor for cell growth and production of violacein. Cultures were dosed with ampicillin in the range of 0.05–0.4 mg mL^-1^ (**Figure 1E**). It was observed that the addition of antibiotic in the range of 0.1–0.4 mg mL^-1^ enhanced violacein production as a stress response, while cell growth was decreased. Ampicillin concentration of 0.1–0.2 mg mL^-1^ maximized violacein production, increasing pigment concentration by 33–36% in comparison to antibiotic-free cultures, without influencing cell growth significantly.

### Violacein Production Under Fed-Batch Fermentation

**FIGURE 2:**
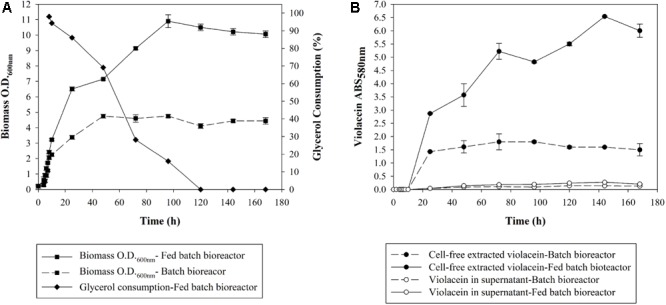
**(A)** Optical density and glycerol consumption of *J. lividum* biomass and **(B)** crude violacein cell-free extracted or leaked in the supernatant produced in bioreactor at 25°C, pH 7, under monitored conditions of ventilation and diluted oxygen.

Aiming at the scale up of violacein production, a 2-L bioreactor was utilized under monitored conditions of pH, temperature, agitation, and ventilation following the optimal conditions found in shake flasks. The fermentation of *J. lividum* carried out in batch mode resulted in maximum biomass concentration of 0.77 mg mL^-1^, with an optical density of 4.75 at 600 nm, after a 48-h incubation as shown in **Figure 2A**. It is notable that the exact same biomass concentration was achieved in shaking flasks. Violacein production was maximized at 24 h reaching the concentration of 0.368 mg mL^-1^ (**Figure 2B**).

Subsequently, in an attempt to increase violacein production, glycerol was added at the logarithmic phase of cell growth in order to increase the carbon source when the dissolved oxygen in the culture fluctuated in low levels ∼20%. Glycerol was chosen because in the optimization study (**Figure 1C**) it induced the higher violacein production compared to glucose and xylose. In **Figure 2A** the glycerol consumption over time is also presented. Biomass is being produced up until 96 h, while glycerol is being consumed until 120 h. With glycerol addition, the ratio of *C*/*N* is increased and therefore violacein production is enhanced as a secondary metabolite. The glycerol addition resulted in biomass production with an optical density of 10.9 at 600 nm at 96 h of incubation, with a satisfactory increase of 2.3-fold in comparison to the batch fermentation. The biomass increase led to 1.828 mg mL^-1^ crude violacein production at 144 h, a fivefold violacein increase compared to the no-glycerol added bioreactor, as shown in **Figure 2B**. The cell-free extracted violacein in the current study was crude, therefore its content was found by measuring the absorbance at 575 nm, applying the extinction coefficient of the pure metabolite found in literature. This way it was estimated that the crude extract consists of 4.5% of pure violacein.

### Dyeing of Polyamide Fabrics

**FIGURE 3:**
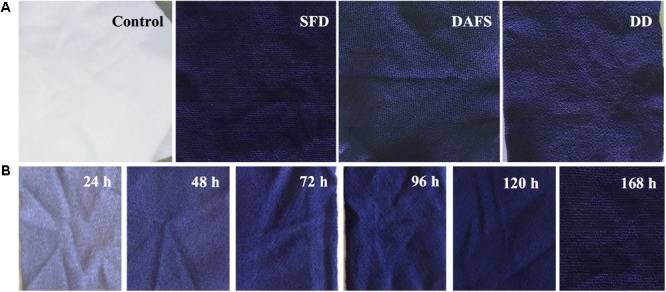
**(A)** Untreated (control) and dyed fabrics via SFD, DAFS, or DD, **(B)** different shades, depending on incubation time, of purple-dyed polyamide 6.6 fabrics via SFD.

Polyamide fabrics were dyed following three different processes, namely SFD, DAFS, and DD (**Figure 3A**). In all cases, samples were acquired and the color coordinates are presented in **Table 1**. Both DAFS and DD dyeing processes at 48 h resulted in fabrics with similar Δ*E* 63.83 and 67.88 and *K*/*S* 7.89 and 10.67, respectively. SFD, however, presents the best dyeing results for 4–6 days of incubation with Δ*E* and *K*/*S* in the vicinity of 71.72–74.81 and 16.48–22.01, respectively. Additionally, it is a notable fact that depending on the incubation time during SFD process, more than one purple hues are acquired (**Figure 3B**). For SFD dyed polyamide samples dyeing strength was evaluated and no alteration of color fastness and staining of dye was indicated at acid and alkaline perspiration or at water (Supplementary Table S1). Abrasion resistance in dry and wet state reached the value 5 which means no staining on the cotton fabric and subsequently no decolorization of the SFD fabrics. Polyamide dyeing with violacein led to poor color fastness after light exposure, when placed 165 h in the XENON arc lamp chamber (Supplementary Table S2 and Supplementary Figure S1).

**Table 1:** Color coordinates of fabrics dyed via SFD, DAFS, and DD processes.

Dyeing process	*L*	*a*	*b*	Δ*E*	*K*/*S*
Control	89.90	0.14	2.09		0.36
SFD^1^	21.31	9.16	–26.39	74.81	22.01
DAFS^2^	34.71	7.75	–29.07	63.83	7.89
DD^3^	30.98	9.06	–30.43	67.88	10.67

Incubation time (h)	SFD				

0	89.90	0.14	2.09	0.36	
24	60.04	3.17	–18.97	36.67	1.26
48	40.71	5.77	–24.53	56.21	4.85
72	30.34	7.71	–26.63	66.55	10.61
96	24.74	8.69	–26.63	71.72	16.48
120	23.1	8.78	–25.47	72.78	18.31
168	21.31	9.16	–26.39	74.81	22.01

*DAFS and DD were conducted for 48 h. SFD results are also presented for different times of incubation. ^*1*^Simultaneous fermentation and dyeing. ^*2*^Dyeing after fermentation and sonication of cell culture. ^*3*^Direct dyeing with cell-free extracted violacein.*

### Antimicrobial Activity of Crude Extract Containing Violacein and Violacein Dyed Polyamide

**FIGURE 4:**
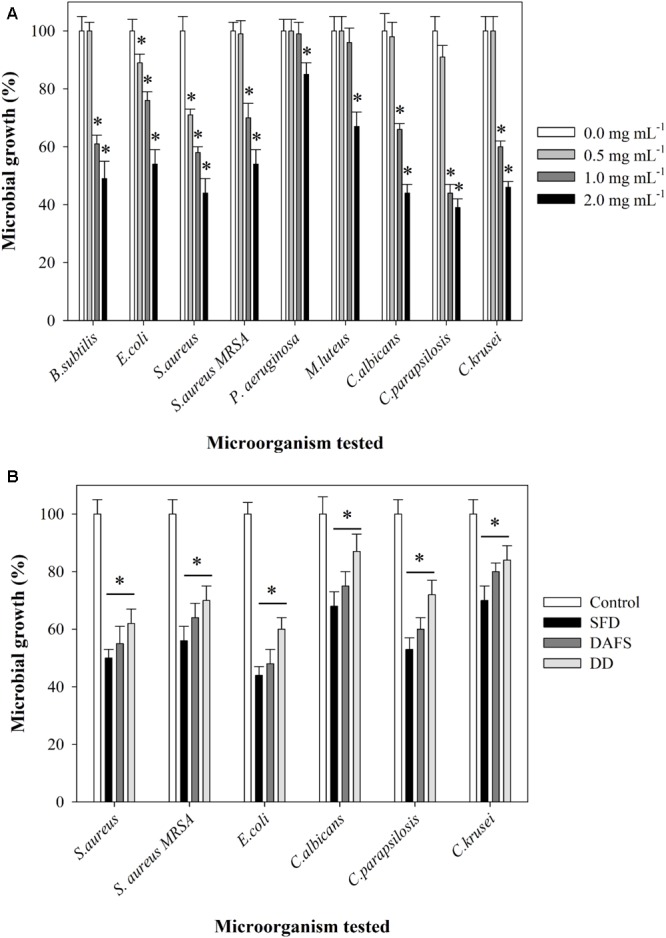
Inhibition of microbial growth by **(A)** cell-free extract containing violacein at concentrations of 0.0, 0.5, 1.0, and 2.0 mg mL^-1^ and **(B)** polyamide 6.6 fabrics dyed via SFD, DAFS, or DD or untreated fabric. Statistical significance (*p* < 0.05) values from control are denoted by asterisks.

**FIGURE 5:**
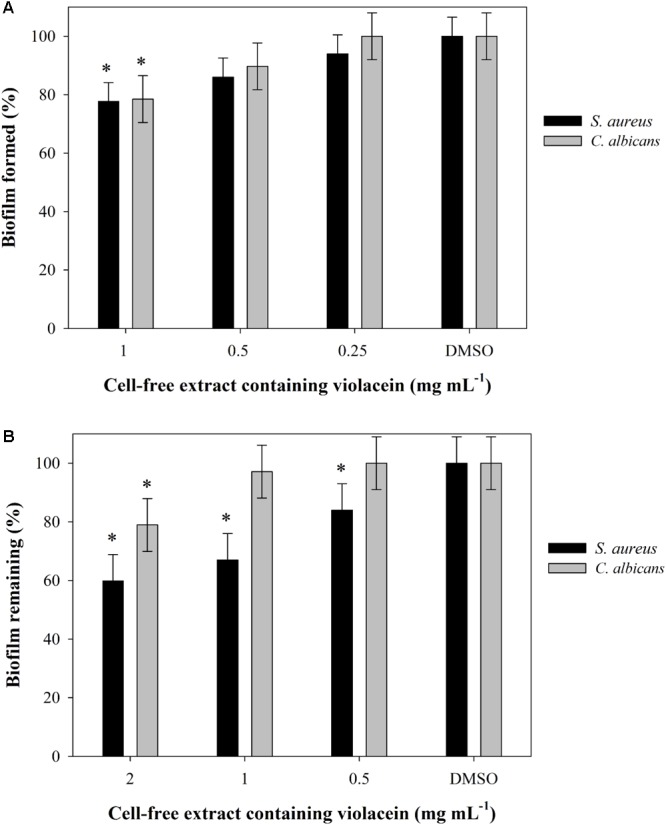
**(A)** Biofilm formation and **(B)** biofilm disruption at different concentrations of cell-free extract containing violacein in DMSO. Statistical significance (*p* < 0.05) values from control are denoted by asterisks.

In the initial screen, crude methanolic extract of *J. lividum* culture was assessed for the antimicrobial properties against two Gram-negative (*E. coli* and *P. aeruginosa*), four Gram-positive (*S aureus, S. aureus* MRSA, *M. luteus*, and *B. subtilis*) bacterial strains, and three fungal strains (*C. albicans, C. krusei*, and *C. parapsilosis*) (**Figure 4A**). Culture extract, containing violacein, was able to inhibit the growth of all tested strains between 15 and 60% in comparison to untreated control at relatively high concentration of 2 mg L^-1^. On the other side, the growth of *S. aureus* was inhibited for 30% even at concentration of 0.5 mg L^-1^. Importantly, *S. aureus* MRSA strain has also been affected with the presence of crude extract containing violacein, although to lesser extent. The least affected was *P. aeruginosa* PAO-1. *C. parapsilosis* was the most sensitive among fungal strains with 55 and 10% growth inhibition at 1 and 0.5 mg L^-1^ of the extract, respectively. Crude methanolic extract of *J. lividum* was also able to moderately inhibit formation and to disperse already formed biofilms of *S. aureus* at extract concentrations of 1 and 2 mg L^-1^, while this effect has not been observed with *C. albicans* (**Figure 5**).

*Staphylococcus aureus* and *Escherichia coli* and three *Candida* strains were used further for the evaluation of the antimicrobial properties of polyamide dyed with this crude bacterial extract containing violacein using three different approaches (**Figure 4B** and Supplementary Figure S2). Violacein dyed polyamide was inhibiting microbial cell growth between 55 and 10%, depending on the strain and on the type of dying procedure. Overall, polyamide dyed using SFD approach showed the greatest antimicrobial effect, while DD approach resulted in the dyed material with the lowest antimicrobial properties. This result correlated well with the intensity of blue dye (**Figure 3A**).

### Antioxidant Activity of Violacein Dyed Polyamide

In this case, the antioxidant activity of dyed fabrics with violacein was investigated with the DPPH radical method. Initially the scavenging ability of crude violacein was examined and an IC_50_ equal to 0.16 mg mL^-1^ was estimated. Subsequently, dyed fabrics through SFD, DAFS, or DD were tested for antioxidant behavior, without demonstrating satisfactory antioxidant results (**Table 2**).

**Table 2:** Scavenging ability of dyed polyamide fabrics with violacein via SFD, DAFS, or DD.

Sample	Scavenging ability (%)
Control	0 ± 0.10
SFD^1^ 24 h	10 ± 0.11
SFD 48 h	12 ± 1.13
SFD 168 h	12 ± 1.07
DAFS^2^ 48 h	8 ± 0.12
DD^3^ 48 h	14 ± 1.9

*^*1*^Simultaneous fermentation and dyeing. ^*2*^Dyeing after fermentation and sonication of cell culture. ^*3*^Direct dyeing with cell-free extracted violacein.*

## Discussion

*J. lividum* is a psychrotrophic species that has been found in glacier and on the skin of amphibians, such as frogs or the red-backed salamander *Plethodon cinereus*, where violacein is produced as bacterial metabolite in concentrations that hinder or kill the pathogen *Batrachochytrium dendrobatidis* (Brucker et al., 2008; Harris et al., 2009; Lu et al., 2009). This pigment’s antimicrobial and antioxidant activity can be exploited for textile dyeing with acquired antimicrobial and antioxidant properties.

Violacein microbial production depends on several factors starting from the microorganism species to the environmental conditions where the microorganism grows. Thus, a culture optimization study is often necessary for improving the production of the certain metabolite. Typically, violacein producing microorganisms are reported in the literature to be cultured in certain media like nutrient or LB broth without further adjustment of the pH. In this study, considering the effect of the initial pH on *J. lividum* growth, pH 7.0 proved to be the most suitable for maximum cell growth and violacein production. Psychrotrophic bacterium RT102 strain, a species very close to *J. lividum*, was grown at pH 6 and 25°C (Nakamura et al., 2003). The optimum temperature for the *J. lividum* growth was found at 25°C, in accordance to literature (Rodrigues et al., 2012; Masuelli et al., 2016) and similar to the *J. lividum* strain IAM13948 (Shirata et al., 2000). On the other hand, a *J. lividum* XT1 strain has been reported to grow better at 15°C, achieving pigment concentration of 0.85 g L^-1^, after 7 days of cultivation (Lu et al., 2009). Concerning the carbon sources tested, inexpensive choices such as glucose, xylose, and glycerol could be derived from exploitation of lignocellulosic biomass and biodiesel waste, respectively, through fermentation procedures (Kumar et al., 2009; Yang et al., 2012). When NB-G, NB-GY, and NB-X were tested as culture media, biomass production was favored, however, there was a negative impact on violacein production, justifying that the carbon source regulates the metabolic path toward violacein production. In particular, *J. lividum* grown in NB-GY medium produced 2.19-fold more violacein comparing to NB-G. In literature the same strain has been grown in LB broth, LB-glucose, and LB-glycerol media. Violacein was produced in the presence of glycerol and to a lesser extent in basic LB, while pigment production was inhibited by glucose (Pantanella et al., 2007). Furthermore, the addition of an antibiotic was examined as a stress inducing factor for cell growth and production of violacein. The ampicillin dosage in the vicinity of 0.1–0.4 mg mL^-1^ enhanced the violacein production up to 36% as a stress response without influencing significantly cell growth. This is in line with the previous report that addition of ampicillin in *J. lividum* culture resulted in higher violacein production up to 25% (Pantanella et al., 2007).

Bacterial violacein is a compound difficult to extract and purify and thus expensive in its commercial form. The scale-up fermentation of *J. lividum* in 2-L bioreactor led to the same biomass concentration, however, 2.7-fold less violacein was produced comparing to shake flasks based on absorbance measurements at 580 nm. At this point, it is important to address that in both flasks and bioreactor experiments, a similar amount of crude extract per volume of fermentation was obtained, contrary to the absorbance measurements that represent violacein concentration. The biomass concentration on the d.w. basis was also comparable, however, possibly due to improved oxygen supply and pH control, and thus less stressful environmental conditions in the bioreactor, violacein production was decreased. Pure violacein comprised the 4.5% of the whole methanol extracted amount, therefore its decrease is not necessarily depicted in the final gravimetric yield of the bioreactor, but it is depicted in terms of optical density at 580 nm. On the other hand, the increase of carbon source through the fed-batch process, led to a notable improvement in violacein production, since the ratio of *C*/*N* is increased considerably triggering the production of violacein. Biomass on d.w. basis and violacein production reached a 2.3- and 5-fold increase, respectively, in comparison to batch fermentation. Violacein production in the fed-batch process also exceeds the small scale violacein outcome in the flasks by 35% in terms of absorbance at 580 nm. In previous studies, the estimation of violacein concentration is derived from spectrophotometric analysis of the extracted crude violacein in ethanol solution and therefore the results presented are not directly comparable. Indicatively, in literature, crude violacein production of 1.75 g L^-1^ has been achieved by an engineered *E. coli* in a 5-L fermentor with interactive control of tryptophan pathway and violacein biosynthetic pathway (Fang et al., 2015). A factorial design has been conducted for the optimization of crude violacein production from *C. violaceum* achieving 21 g L^-1^ biomass and 0.43 g L^-1^ crude violacein production (Mendes et al., 2001). A psychrotrophic bacterium RT102 strain culture in a 3 L fermentor reached maximum biomass yield of 15 g L^-1^ and violacein concentration 3.5 g L^-1^ at 30 h. However, in this case, the violacein was extracted from the supernatant (Nakamura et al., 2003). Moreover, a pigment content of 0.8 g g^-1^ of dry biomass has been achieved by a *J. lividum* XT1 strain through a culture optimization study (Lu et al., 2009).

Bacterial violacein has been utilized before for dyeing different kind of textiles (Choi et al., 2015b). The microbial pigment is transported from the aqueous phase and absorbed onto particular reactive groups of the fabric. In our study, the fabrics dyed via SFD, DAFS, or DD attained similar *K*/*S* and Δ*E* that were quite impressive. On the other hand, the fabrics that underwent SFD process obtained even darker color and better antimicrobial properties (**Figures 3A, 4B**). This result is of outmost importance, since *J. lividum* under SFD could provide outstanding dyed textiles with the minimum environmental impact, since no chemical solvents were used, while maintaining a mild fermentation process. This way textiles could be dyed via SFD and the extracted violacein could be utilized even for different applications as in biomedicine after further purification. Furthermore, the dyed fabrics presented poor light fastness. Obviously, high resistance to degradation or fading of fabric dyes and prints due to light is an important requirement for clothing items, but becomes less important in different applications restricted to the indoor use of textiles, such as medical applications. For fabrics dyed via SFD process, sterilization and washing steps are essential for neutralizing the microbial load, aiming at a final product that meets the market requirements for a specific application (e.g., textile industry, medicine, etc.). In literature, polyamide fabrics have been dyed via immersing in violacein solution in several organic solvents or in the autoclaved culture supernatant for 20 min, however, no color coordinates were provided for the comparison with the present study (Shirata et al., 2000). Herein, through the SFD process a feasible biotechnological procedure of fabric dyeing is proposed by using the crude extract that contains the violacein, without using organic solvents. This way decrease of the process cost and the environmental impact can be achieved.

Textiles have evolved from being just commodity materials to providing an array of benefits, including additional comfort and protection, improved hygiene, and supporting healthcare. The textile industry has shown great interest toward the manufacturing of smart multifunctional fabrics oriented for medical applications (Carosio and Monero, 2004; Marques et al., 2014; Armstrong et al., 2017). In particular, there is great interest in obtaining fabrics with antimicrobial properties capable of controlling the spread of multiresistant microorganisms. In literature, several studies have been carried out for acquiring textiles with antimicrobial activity via bonding of phenols, formaldehyde derivatives and others, however, the toxicity and no-biodegradability of these compounds renders them non-sustainable (Gupta and Bhaumik, 2007; Iyigundogdu et al., 2017). Violacein and other natural pigments have been examined for application in textile industry as they bring no harmful effects on environment and aquatic ecosystem and provide more developed functionalities to textiles simultaneously (Yusuf et al., 2017). Violacein has been reported to have antibacterial activity against several microorganisms such as *S. aureus, B. licheniformis, B. subtilis, B. megaterium*, and *P. aeruginosa*, as well as antifungal activity against *Colletotrichum dematium, Fusarium solani f.* spp. *mori, Bipolaris leersiae a.o.* (Shirata et al., 2000; Nakamura et al., 2003; Cazoto et al., 2011; Martins et al., 2011). We have confirmed this broad spectrum of antibacterial properties of methanol culture extract containing violacein. Minimum inhibitory concentrations of pure violacein against different microbial strains vary from 2 μg mL^-1^ to more than 50 μg mL^-1^, which is in line with our study since we have shown that the content of violacein in the extract was about 5%. Importantly, violacein dyed polyamide showed excellent antimicrobial properties, both against bacteria and *Candida* strains. Notably, SFD fabric rendered the best antimicrobial properties. Particularly the SFD fabrics mitigate cell growth of *S. aureus* and *S. aureus* MRSA by 50 and 44%, compared to the 56 and 46% bacterial reduction that crude violacein exhibits at the concentration of 2 mg mL^-1^. However, this is not the case for the *E. coli* and *Candida* strains for which even SFD fabrics present less inhibitive effect as opposed to cell-free extracted crude violacein. In literature there in no documentation of conducting antimicrobial tests on treated fabrics with violacein. Researchers have prepared antimicrobial PA 6.6 fabrics with silver nanoparticle coating after plasma treatment. Indicatively, 20 and 100 nm sized AgNPs polyamide fabrics induced 100 and 20% bacterial reduction of *E. coli* and 82 and 19% of *S. aureus*, respectively (Zille et al., 2015). In fact the use of metal nanoparticles in fabrics is a widely used technique for the preparation of antimicrobial fabrics (Humberto, 2015), however, in the herein work a more eco-friendly approach is suggested. Cotton fabrics coated with chitosan have presented reduction of 76–96% of *S. aureus* and 72–94% of *E. coli* growth (Rahman Bhuiyan et al., 2017). Another study included the enhancement of antibacterial properties of cotton fabrics by coating herbal nanoparticles from *Azadirachta indica* resulting in 81% reduction of *E. coli* and 77% of *S. aureus*, respectively (Subramani et al., 2017).

When it comes to textiles with the potential to be used in medical applications, antioxidant properties are essential along with their antimicrobial profile, especially in the case of “cosmetotextiles” (Zemljič et al., 2014). Antioxidants are natural substances used to regulate the processes triggered by external aggressions, thereby preventing the oxidative stress. Violacein possesses antioxidant properties based on the density functional theory method (Cao et al., 2007). In short, it was calculated that a certain N–H bond of violacein structure has bond dissociation energy (BDE) slightly lower than that of the N–H in DPPH–H, therefore, violacein can scavenge DPPH radicals. Additionally, violacein antioxidant properties have been justified before via reacting with DPPH, nitric oxide, superoxide radicals, and decreasing the hydroxyl radical electron paramagnetic resonance (EPR) signal. In this case, the antioxidant activity of crude violacein and dyed fabrics with violacein was investigated with the DPPH radical method. Crude violacein presented an IC_50_ equal to 0.16 mg mL^-1^, expecting that also other chemical components of the crude extract contribute to the antioxidant behavior. Via DPPH tests on pure violacein reported previously (Konzen et al., 2006), it was concluded that pure violacein exhibits an IC_50_ equal to 0.087 mg mL^-1^, 1.8-fold lower than crude violacein, as expected, after incubation for 1 h at 37°C. The antioxidant ability of polyamide textiles dyed with violacein has not been examined before, so the direct comparison is not possible. Nonetheless, indicatively, the treatment of cotton fabrics with gallnut extract resulted in 100% scavenging activity of DPPH radicals (Hong, 2014). On the contrary, the preparation of cotton fabrics coated with chitosan that contains biologically active antioxidant substances by Viola Tricolor did not present any scavenging activity (Mocanu et al., 2013).

An eco-friendly approach is proposed for synthetic fabrics dyeing using violacein from *J. lividum*. Pigment production was improved and scaled up in bioreactor fermentation, aiming at the effective dyeing of textiles with antifungal and antibacterial properties. For the efficient application of the microbial dye on polyamide fabrics, three different processes were evaluated based on color characteristics and antimicrobial properties. The SFD process showed the greatest antimicrobial effect providing outstanding dyed textiles with the minimum environmental impact, since extraction and purification steps of violacein were avoided. Further studies are needed to evaluate the potential of these functional textiles to support healthcare for medical applications through the control of multiresistant microorganisms. Moreover, violacein production can be examined using lignocellulosic biomass and biodiesel waste as carbon source for *J. lividum* growth in order to render the process more sustainable. Also the encapsulation of violacein for controlled release on the fabric is of great interest and experiments are being carried out at the moment toward this direction.

## Author Contributions

MKan, MM, MKal, and SV carried out the experiments and wrote the manuscript. MKan and MKal contributed in the optimization and scale up of violacein production. SV performed the characterization of the dyed textiles. MM carried out the antimicrobial experiments. JN-R, DK, and ET participated in study conception, data interpretation, and corrected the manuscript. All authors have read and approved the final manuscript.

## Conflict of Interest Statement

The authors declare that the research was conducted in the absence of any commercial or financial relationships that could be construed as a potential conflict of interest.
