# Supplementary material for: Microbial Production of Violacein and Process Optimization for Dyeing Polyamide Fabrics With Acquired Antimicrobial Properties
Source: Front Microbiol. 2018 Jul 10;9:1495. doi: 10.3389/fmicb.2018.01495 (PMC6048185; doi:10.3389/fmicb.2018.01495)
Supplement: Supplementary file 1 [file Data_Sheet_1.PDF]

frontiers

Supplementary Material

Microbial Production of Violacein and Process Optimization for Dyeing Polyamide Fabrics with Acquired Antimicrobial Properties

Maria Kanelli, Mina Mandic, Margarita Kalakona, Sozon Vasilakos, D. Kekos, Jasmina Nikodinovic-Runic, Evangelos Topakas*

*** Correspondence:** Evangelos Topakas: vtopakas@chemeng.ntua.gr

Table S1: Color fastness of SFD fabrics tested against acid and alkaline perspiration and washing with water according to EN ISO 105-E04[1] and EN ISO-E01[2]. Evaluation for staining on a gray scale from 1 to 5, where 5 is regarded as no staining.

	color change	wool	polyacrylic	polyester	polyamide	cotton	acetate
Acid perspiration[1]	5	5	5	5	5	5	5
Alkaline perspiration[1]	5	5	5	5	5	5	5
Washing with water[2]	5	5	5	5	5	5	5

Table S2: Color change of SFD fabrics after exposure to artificial light for 165 h according to EN ISO 105-B02:2013.

	L	a	b	ΔE	K/S
Unmodified	89.90	0.14	2.09		0.36
SFD 24h[1]	50.48	5.23	-26.65	49.05	2.6
SFD 24h[2]	70.67	0.45	-11.14	23.34	0.53
SFD 96h[1]	24.3	8.46	-25.32	71.58	16.33
SFD 96h[2]	24.74	8.69	-26.63	30.22	1.01

[1]Before exposure to artificial light, [2]After exposure to artificial light

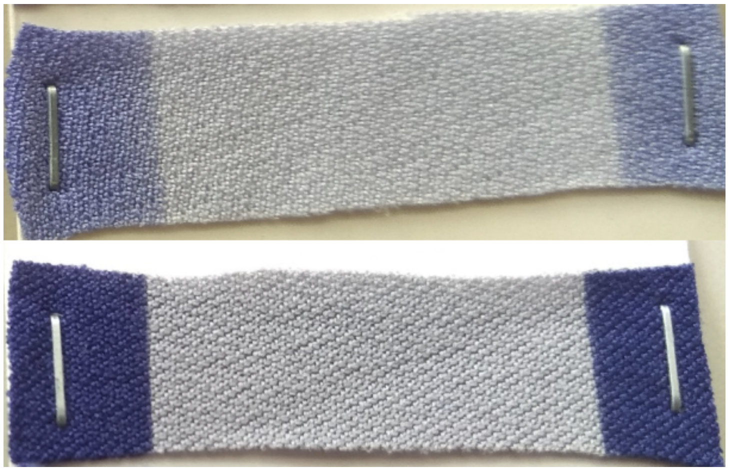

Figure S1: Light fastness of SFD fabrics, dyed for 24 h and 96 h, to artificial XENON light according to EN ISO 105-B02:2013.

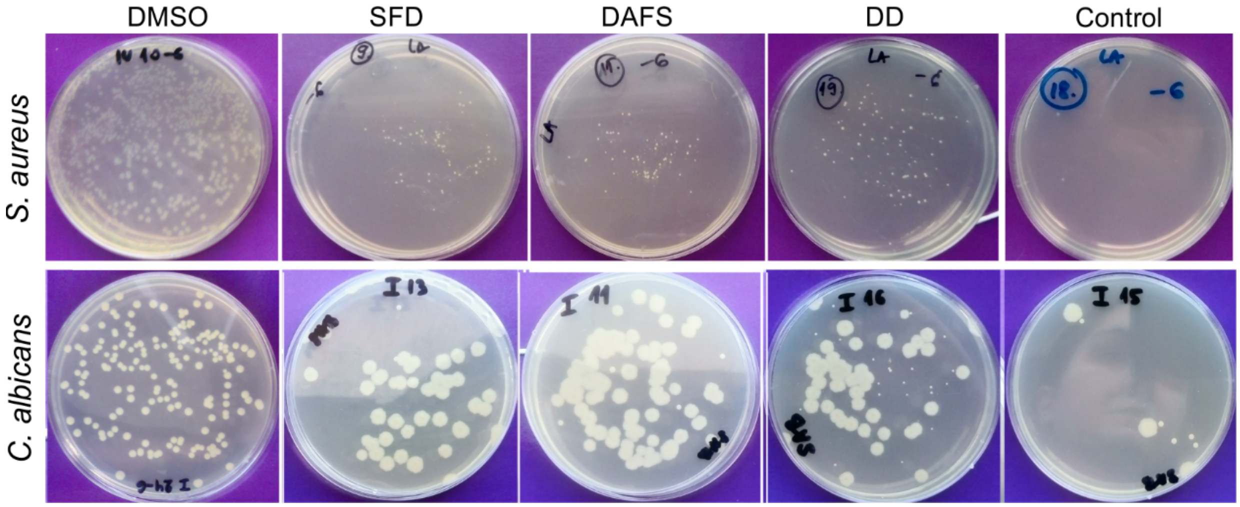

Figure S2: Colony forming units (CFU) counts, after *S. aureus* and *C. albicans* cultures were exposed to PA dyed materials via SFD, DAFS and DD, including DMSO and sterilized control material in medium without inoculum.
